# STRATEGIES USED BY OHIO’S NURSING HOMES TO RETAIN CERTIFIED NURSING ASSISTANTS: DO THEY WORK?

**DOI:** 10.1093/geroni/igad104.0978

**Published:** 2023-12-21

**Authors:** Oksana Dikhtyar, Ian Matt Nelson

**Affiliations:** Scripps Gerontology Center, Miami University, Oxford, Ohio, United States; Miami University, Oxford, Ohio, United States

## Abstract

A low retention rate of certified nursing assistants (CNA) in nursing homes (NHs) is a known problem; it drives up facilities’ operating costs and negatively impacts the quality of care provided. The COVID-19 pandemic exacerbated this problem and left facilities scrambling to retain workers. Using data from the 2021 Ohio Biennial Survey of Long-Term Care Facilities, this study examines strategies implemented by facilities to improve retention of their CNAs. Facilities have tried to implement a variety of workplace environment changes such as allowing staff teams to manage schedule and financial benefit strategies such as longevity wage increase to improve their CNA retention. The statewide average NH retention rate was 64% for CNAs ranging between 0 and 100%. Three groups were constructed from the retention data: high or rate of 75% or higher (N = 220), medium or between 50% and 75% (N = 254), and low or under 50% (N = 145). We found that a higher proportion of low-retention facilities implemented most of these strategies compared to facilities with high and medium CNA retention rates. At the same time, we found that a higher proportion of administrators in the high retention group knew all their CNAs by name compared to the low-retention group (50.0% vs 41.7%). These findings suggest that environmental and financial strategies may not have much of an impact on retention rates, but making people feel respected and appreciated could.

